# Relationship between Serum Homocysteine Concentration and Dietary Factors in Young Japanese Women

**DOI:** 10.3390/nu15224740

**Published:** 2023-11-10

**Authors:** Akiko Tajima, Yoshinori Kubo, Sayaka Horiguchi, Kumiko Shoji, Terue Kawabata

**Affiliations:** 1 Faculty of Nutrition, Kagawa Nutrition University, 3-9-21 Chiyoda, Sakado 350-0288, Saitama, Japan; shori@saitama-med.ac.jp (S.H.); shoji.kumiko@eiyo.ac.jp (K.S.); kawabata@eiyo.ac.jp (T.K.); 2 Division of Anatomy and Cell Biology, Department of Anatomy, Shiga University of Medical Science, Seta Tsukinowa-cho, Otsu 520-2192, Shiga, Japan; kubo11@belle.shiga-med.ac.jp

**Keywords:** homocysteine, dietary fiber, soluble fiber, insoluble fiber, fruits, mushrooms, vitamin B_6_, vitamin B_12_, folate

## Abstract

Homocysteine is a methionine metabolism intermediate and its increased blood levels are associated with a higher risk of noncommunicable diseases. Reportedly, blood homocysteine levels increase with inadequate folate, vitamin B_6_, and vitamin B_12_ intake; however, its relationship with dietary factors other than these three vitamins remains unknown. Thus, we investigated the relationship of homocysteine with other nutrient intake. We performed a dietary survey on 227 young women using a food record with approximate amounts for 7 consecutive days in conjunction with digital imaging. We collected early morning fasting blood samples the day after the dietary survey was completed and analyzed the serum homocysteine levels. We observed that the serum homocysteine concentrations were significantly negatively associated with soluble, insoluble, and total fiber intake. In addition, participants with high fruit and mushroom intake displayed lower serum homocysteine concentrations, suggesting dietary fiber involvement from these foods. However, we observed no serum homocysteine concentration-related association with cereals and vegetables (well-documented dietary fiber sources) or with fruits and mushrooms. In conclusion, fiber quality-related differences could thus be caused by different sources, including antioxidant components such as fruit polyphenols and mushroom antioxidant and anti-inflammatory factors.

## 1. Introduction

Although infectious diseases, such as pneumonia and tuberculosis, were the main causes of death among Japanese people during and after World War II, the main causes of death among Japanese people today are noncommunicable diseases (NCDs), such as cancer and heart and cerebrovascular diseases [1]. Approximately 82% of total deaths are reportedly caused by NCDs [2], so preventive strategies are urgently needed. According to the World Health Organization (WHO) definition, NCDs is a collective term for chronic diseases, including cancer, diabetes, cardiovascular disease, respiratory disease, and mental illness, which have various causes such as unhealthy diets, lack of exercise, smoking, excessive alcohol consumption, and air pollution.

In 1969, McCully found that patients with hyper homocystinuria developed atherosclerotic thrombotic lesions at a young age [3]. He noted that atherosclerotic plaques scattered throughout the arteries of many organs were characteristic of patients with homocystinuria and suggested that elevated blood homocysteine levels may be a factor in the development of atherosclerosis [4]. Since that report, the association between homocysteine and atherosclerosis has gained attention. A number of epidemiological studies have shown that elevated blood homocysteine levels are an independent risk factor for coronary ischemic disease, stroke, peripheral vascular disease, and venous thrombosis [5,6] and are associated with an increased risk of NCDs.

**Figure 1:**
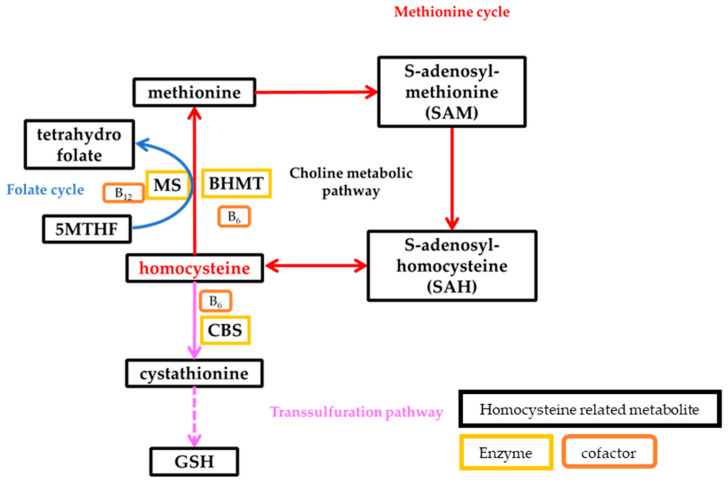
Factors associated with the methionine cycle, folate cycle, and transsulfuration pathway in homocysteine metabolism. Abbreviations: BHMT, betaine–homocysteine methyltransferase; MS, methionine synthase; CBS, cystathionine *β*-synthase; 5MTHF, 5-methyltetrahydrofolate; GSH, glutathione.

Homocysteine is produced in the body by metabolizing methionine, an essential amino acid [7]. Homocysteine metabolism depends on folate and methionine metabolism and is mediated by remethylation via betaine–homocysteine methyltransferase (BHMT) and methionine synthase (MS) and by the transsulfuration pathway via cystathionine betasynthase (CBS). The homocysteine metabolic pathway involves cobalamin (B_12_) and pyridoxine (B_6_) as cofactors [8] (Figure 1).

Factors reported to be related to serum homocysteine concentrations include blood folate concentration, folate intake, vitamin B_6_ and vitamin B_12_ intake, smoking, coffee intake, and physical activity [7,9,10,11]. However, it is unclear if nutrients other than folate and vitamins B_6_ and B_12_ are associated with homocysteine metabolism. Therefore, this study aims to examine dietary factors that decrease blood homocysteine levels, focusing on nutrients other than folate and vitamins B_6_ and B_12_, which are clearly associated with homocysteine metabolism. It is thought that examining factors that reduce blood homocysteine levels could contribute to primary prevention of various diseases and lead to a decrease in NCDs.

## 2. Materials and Methods

### 2.1. Study Design

The survey was conducted between October and December 2018, and a cross-sectional study design was used. A survey on lifestyle was conducted on the 1st day of the study, and a dietary survey using a standard amount recording method and a physical condition check survey were conducted from the 2nd to the 8th days of the study. On the morning of the 9th day, which was the day after the end of the 7 consecutive days of food recording and anthropometric and blood pressure measurements, fasting blood samples were taken.

### 2.2. Subjects

Healthy young women between the ages of 18 and 25 were included in the study. The exclusion criteria for these women were a history of or current serious hepatic, renal, cardiac, pulmonary, gastrointestinal (including gastrectomy) disorders, organ disorders, diabetes, food-allergy disease, or other serious diseases; use of drugs that may affect the laboratory values measured in this study (those associated with lipid metabolism, folate metabolism, anti-inflammatory or antioxidant responses); pregnant or nursing; and planning to become pregnant. We also excluded women deemed unsuitable by the medical doctor. A total of 258 individuals were asked to participate in the study, and informed consent to participate was obtained. After the study started, due to withdrawals and declinations of consent, the final number of participants in the study was 227.

### 2.3. Lifestyle Survey and Physical Condition Check Survey

The lifestyle survey included items related to exercise habits, smoking status, and eating habits. The physical condition check survey was conducted daily during the dietary survey period and included a survey of the patient’s physical condition on that day, use of pharmaceuticals, unusual diet or exercise, regularity of menstrual cycles, and number of days.

### 2.4. Dietary Survey

The dietary survey was conducted for 7 consecutive days using the approximate-quantity recording method in conjunction with digital images from a digital camera or smartphone. The authors requested that meals during this time be served as usual. All meals or foods except water were photographed, and at the same time, the subjects were asked to fill in the names of the dishes, ingredients used, and approximate amounts on the meal record form. When taking photographs, we requested that one photo be taken from directly above the food and one from a diagonal angle, a post-meal photo be taken, and a designated card be placed at the corner of the meal tray or in place of the object to serve as a ruler.

The authors used digital images and dietary records to estimate the type and weight of individual foods consumed by the subjects. The consistency between the dietary record and the digital image was checked, and if the dietary record and digital image did not match, subjects were asked to confirm. The Dietary Survey Manual [12] and the Energy Quick Guide [13] were used as reference materials for estimating food weights. Excel eiyoukun Version 8.2 (Kenpakusha, Tokyo, Japan) was used to calculate the energy and nutrient intakes. When calculating the energy and nutrient intakes, food items that were considered closest to the actual dietary situation were selected according to Japan’s Standard Tables of Food Composition, 2015 [14], and if the appropriate number could not be determined from the dietary records and digital images alone, the selection was based on the Dietary Survey Manual [12]. For food service, prepared foods, and commercial products, the analysis was performed by replacing similar food items or combining multiple food numbers to approximate the company’s published Nutrition Facts label. In cases in which the company did not publish the nutritional information, the same items consumed by the subject were purchased, and the ingredients used were weighed and recorded for analysis. When not available for purchase, the Calorie Guide [15,16,17] was consulted and replaced with a similar dish. Seasoning percentages were taken from the Dietary Survey Manual, and oil absorption rates for fried foods were taken from Basic Data for Cooking [18]. Excel eiyoukun Version 8.2 was also used to convert food numbers to food groups and precooked weight to cooked weight. Nutrient intake was defined as intake per 1000 kcal.

### 2.5. Anthropometric Measurement

Height, weight, body mass index (BMI), body fat percentage, and body fat mass were measured. Body weight, body fat percentage, and body fat mass were measured by the impedance method using the TBF-110 (TANITA, Tokyo, Japan). An arm ES-P2000 Electronic Blood Pressure Monitor (TERUMO, Tokyo, Japan) was used to measure blood pressure twice after 1 to 2 min of rest in the sitting position, and the average value was obtained.

### 2.6. Blood Drawing

The day before the blood draw, the subject was instructed to finish dinner by 10:00 p.m. and then fast for 10 h. During the fasting period, the patient was instructed to consume only water and tea. If the fasting time was <10 h, the date and time of blood collection were adjusted.

### 2.7. Blood Analysis

Blood was left at room temperature for approximately 30 min after collection, and after confirmation of coagulation, the serum was separated by centrifugation at 2000 rpm and 4 °C for 10 min. The serum was aliquoted into PP tubes and stored in a −80 °C freezer until analysis. Serum homocysteine concentrations were analyzed by liquid chromatography–tandem mass spectrometry [19].

### 2.8. Ethics Approval

Written consent to participate in this study was obtained from the subjects. If the subject was a minor, written consent was also obtained from the parent or guardian. This study was approved by the Research Ethics Review Committee of Kagawa Nutrition University (protocol code no. 204).

### 2.9. Statistical Analysis

Normality was confirmed by performing the Shapiro–Wilk *W*-test. In the case of a nonnormal distribution, a Box–Cox transformation was performed to make the distribution as normal as possible. The mean ± standard deviation values were reported for normal distributions, and the median and 25th and 75th percentile values were reported for nonnormal distributions. Multiple regression analysis was performed with serum homocysteine concentration as the objective variable and nutrient intake as the explanatory variable. Analysis of covariance was performed with the intake by food group divided into tertiles (T1, T2, and T3) and the serum homocysteine concentration as the objective variable. Factors that were significant were subjected to multiple comparisons. Adjustment factors for Model 1 were age, BMI, menstrual cycle, birth control pill use, and physical activity. To those Model 1 factors, Model 2 added vitamin B_6_ and vitamin B_12_ intake, and Model 3 added folate intake to the factors used in Model 2.

All statistical analyses were performed in JMP Pro ver. 16 (SAS Institute Inc., Cary, NC, USA). Two-sided *p*-values < 0.05 were accepted as indicating significance.

## 3. Results

### 3.1. Target Characteristics

Table 1 shows the subjects’ characteristics. The median (25th, 75th percentiles) age of the subjects was 20 (19, 21) years, and the mean ± standard deviation of BMI was 20.3 ± 1.9 kg/m^2^ and body fat percentage was 25.4 ± 4.2%. The median (25th, 75th percentiles) systolic and diastolic blood pressures were 107 (100, 115) mmHg and 69 (64, 74) mmHg, respectively, and serum homocysteine concentration was 6.39 (5.53, 7.41) µmol/L.

### 3.2. Relationship between Energy/Nutrient Intake and Serum Homocysteine Concentration

The relationship between energy/nutrient intake and serum homocysteine concentration is shown in Table 2. The median (25th, 75th percentile) energy and nutrient intakes were 1729 (1454, 1961) kcal/day, with the percentages of total energy being 14% protein, 32% fat, and 54% carbohydrate.

Regarding the relationship between serum homocysteine concentrations and nutrient intake, a significant negative association was found between protein, total dietary fiber, soluble fiber, insoluble fiber, vitamin A, vitamin E, vitamin K, vitamin B_2_, vitamin B_12_, vitamin C, folate, potassium, calcium intake, and serum homocysteine concentration in Model 1, adjusted for age, BMI, menstrual cycle, birth control pill use, and physical activity. Model 2, which added vitamin B_6_ and vitamin B_12_ intake to the adjustment factors in Model 1, showed a significant negative association between protein, total dietary fiber, soluble fiber, insoluble fiber, vitamin K, vitamin C, folate, potassium, calcium intake, and serum homocysteine concentration. However, the associations with vitamin A, vitamin E, and vitamin B_2_ intake that were associated with serum homocysteine concentrations in Model 1 disappeared. In Model 3, in which folate intake was added to the adjustment factors in Model 2, significant negative associations were found only between serum homocysteine concentration and total dietary fiber, soluble fiber, and insoluble fiber. However, the associations of protein, vitamin K, vitamin C, potassium, and calcium intake with serum homocysteine concentrations in Model 2 disappeared.

### 3.3. Relationship between Food Group Intake and Serum Homocysteine Concentration

**Figure 2:**
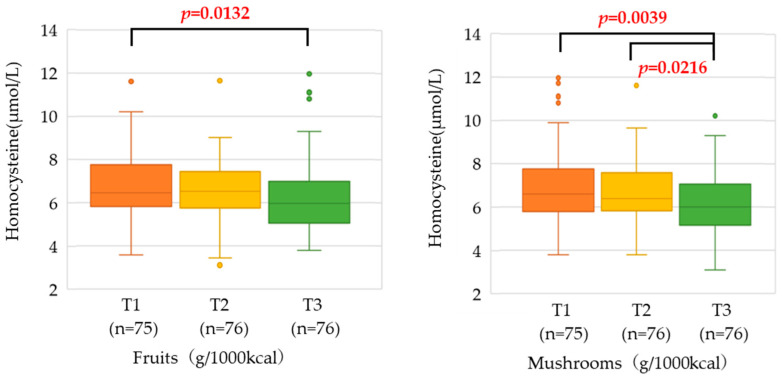
Association with serum homocysteine concentration by fruit and mushroom intake tertile. After analysis of covariance, only significant factors were subjected to multiple comparisons.

The relationship between intake by food group and serum homocysteine concentration is shown in Table 3. The results showed that the higher the intake of fruits and mushrooms, the lower the serum homocysteine concentration (*p* = 0.0060 and *p* = 0.0091, respectively). Further multiple comparisons were made among three groups, which are T1, T2, and T3. In fruits, the T1 had significantly higher serum homocysteine than T3 (*p* = 0.0132). In mushrooms, T1 and T2 had significantly higher serum homocysteine than T3 (*p* = 0.0039, 0.0216, respectively) (Figure 2). No association was found between serum homocysteine concentrations and food groups other than fruits and mushrooms, such as cereals, vegetables high in beta-carotene, other vegetables, and legumes.

### 3.4. Dietary Fiber Intake by Food Group

Dietary fiber intake (mg/1000 kcal) by food group is shown in Table 4. The highest total fiber intake came from cereals, followed by fiber from other vegetables and green vegetables. The total fiber intake from other vegetables and green and yellow vegetables together exceeded the total fiber intake from cereals. There was no difference in total fiber intake from potatoes, fruits, and legumes. The same results as those obtained for total dietary fiber were obtained for soluble and insoluble dietary fiber.

## 4. Discussion

This study focused on dietary nutrients other than folate, vitamin B_6_, and vitamin B_12_, which are clearly associated with serum homocysteine concentrations, and other dietary components, such as insoluble fiber, that reduce blood homocysteine concentrations. The results showed that soluble fiber, insoluble fiber, and total fiber were negatively associated with serum homocysteine concentrations. Among foods, subjects with higher intake of fruits and mushrooms had lower serum homocysteine concentrations. As far as we can tell, there have been no studies in young women on the relationship between serum homocysteine concentrations and various nutrients and food groups, removing the effects of vitamin B_6_, vitamin B_12_, and folate intake as we have done.

Vitamin B_6_, vitamin B_12_, and folate all have a direct relationship with homocysteine metabolism. Vitamin B_6_ is the cofactor of CBS, which metabolizes homocysteine to cystathionine, and is also a cofactor of BHMT, which metabolizes homocysteine to methionine. In addition, folate and vitamin B_12_ are essential vitamins for MS in the metabolism of homocysteine to methionine. Therefore, many studies have previously reported that vitamin B_6_, vitamin B_12_, and folate intake affect homocysteine metabolism [7,9,20]. In the present study, the relationships between serum homocysteine concentrations and nutrient and food group intake disappeared for most nutrients and food groups, except for soluble fiber, insoluble fiber, total dietary fiber, and fruit and mushroom intake as vitamin B_6_, vitamin B_12_, and folate intake were progressively added as adjustment variables. Even vitamin K, vitamin C, potassium, and calcium, which showed associations similar to those of vitamin B_12_ and folate in Model 1, lost their associations with serum homocysteine concentrations in Model 3, in which folate was added as an adjustment variable. This finding suggests that the relationships between serum homocysteine concentrations and vitamin B_6_, vitamin B_12_, and folate are extremely strong, as in previous studies.

Previous studies have reported that dietary fiber is involved in anti-inflammatory effects. The mechanism suggests that short-chain fatty acids [21], produced by fermentation of dietary fiber by intestinal bacteria, improve glucose and lipid metabolism and are involved in the regulation of inflammatory responses [22]. Fiber also improves insulin resistance by slowing the evacuation rate of gastric contents and decreasing the rate of carbohydrate digestion and absorption. In addition, it increases fecal excretion of cholesterol and improves lipid and lipoprotein metabolism. These actions are thought to be effective in reducing inflammation [21], including an anti-inflammatory effect of dietary fiber, which may then lower serum homocysteine levels. Our study results showed that soluble fiber, insoluble fiber, and total fiber were negatively associated with serum homocysteine concentration after adjustment for vitamin B_6_, vitamin B_12_, and folate. Therefore we speculate that the beneficial effects of dietary fiber in reducing inflammation may have caused the decrease in serum homocysteine levels.

Since fruits and mushrooms are sources of dietary fiber, it is possible that the dietary fiber in these foods was responsible for reducing the serum homocysteine levels. However, no association has been found between cereals and vegetables, which contain fiber (as does fruit), and serum homocysteine concentrations. The observed association of serum homocysteine levels with fruits but not with cereals or vegetables may be related to differences in the quality of fiber from different sources and to antioxidant and anti-inflammatory components present in fruits but not in cereals or vegetables. Previously, a UK cohort that examined the association between dietary fiber from cereals and cardiovascular disease risk factors found a significant negative association between dietary fiber from cereals and serum homocysteine concentrations [23]. However, that study did not consider the effects of vitamin B_6_, vitamin B_12_, and folate on homocysteine metabolism. Since cereals are a source of vitamin B_6_ and folate [24], it is possible that these nutrients in cereals are associated with an inverse correlation between serum homocysteine concentration and dietary fiber consumed from cereals. A French cohort study examined the association between cardiovascular disease risk factors and dietary fiber sources, which revealed a significant association between dietary fiber from cereal grains and plasma homocysteine concentrations [25]. The dietary fiber intake in the previous study was 6.4 g/1000 kcal, similar to the results of this study, as cereals are the main source of dietary fiber. However, the primary source of dietary fiber in Japan is reported to be white rice [26], followed by bread. In contrast, in France, the primary source is baguette. This difference in cereal grain sources could have resulted in variations in the type of fiber consumed, and thus, no association was found between cereal grains and serum homocysteine concentrations in our study. Another UK cross-sectional study showed negative associations with many clinical markers, such as *C*-reactive protein (CRP), BMI, body fat percentage, and abdominal circumference, in dietary fiber consumed from fruits, with a higher number of associated clinical markers than those in vegetables [27]. The authors stated that the mechanism may be related to changes in the intestinal microbiota in response to various types of dietary fiber, such as cellulose and lignin, combined with bioactive compounds, such as polyphenols.

Pectin is the most abundant dietary fiber in fruits [28]. A previous study examined the relationship between pectin and plasma homocysteine concentration by feeding rats with either citrus pectin or cellulose. The results showed that rats fed with citrus pectin had higher plasma folate concentrations and lower plasma homocysteine concentrations than those fed with cellulose [29]. Therefore, there is a possibility that the pectin present in fruits may have altered blood folate concentrations, indicating an association between fruits and serum homocysteine concentrations. Fruits are a source of vitamin C, which is known for its antioxidant properties. A cross-sectional study revealed a negative correlation between plasma homocysteine and plasma vitamin C concentrations, suggesting that vitamin C scavenges free radicals formed by homocysteine and may be associated with plasma homocysteine concentrations [30]. In this study, the results also may have been influenced by differences in the quality of antioxidants and dietary fiber in fruits and vegetables.

The highest associations with health effects among the food groups in this study were observed for mushrooms, which have many beneficial properties (including antioxidant, antibacterial, antiviral, anticancer, and anti-inflammatory effects) that are known to improve cardiovascular function [31]. Several compounds in mushrooms can inhibit the synthesis of inflammatory mediators, such as cytokines, interleukins, prostaglandins, nitrogen oxides, and CRP, through inhibition of signaling pathways associated with the NF-κB nuclear receptor [31]. Polysaccharides that are particularly essential for strengthening human immunity and modulating defense responses are components of mushroom mycelia and include glucan, chitin, and chitosan [32], compounds that are dietary fiber and protect the intestinal mucosa [33,34]. Therefore, the combination of mushrooms’ beneficial properties (antioxidant, antibacterial, antiviral, anticancer, anti-inflammatory, and immune-enhancing effects related to dietary fiber) may have been associated with serum homocysteine levels. The dietary fiber found in mushrooms is β-glucan [35]. Although β-glucan is also found in cereals and algae, its structure varies depending on the food source [36]. β-glucan from mushrooms has immunostimulatory properties, whereas β-glucan from shiitake mushrooms is used as an antineoplastic agent (lentinan). Lentinan has been shown to act on reduced macrophages and inhibit oxidized macrophages. Oxidized macrophages produce reactive oxygen intermediates, tumor necrosis factor, and interleukin-6 (IL-6) [37]. Although the relationship between β-glucan in mushrooms and serum homocysteine concentration is unknown, a previous study that examined the relationship between several inflammatory biomarkers associated with atherosclerosis risk showed a significant positive association between IL-6 and blood homocysteine concentration [38]. Therefore, β-glucan in mushrooms may have also affected serum homocysteine levels by suppressing oxidized macrophages, and subjects with a higher mushroom intake may have had lower serum homocysteine levels.

Among the previous studies examining the relationship between serum homocysteine concentrations, nutrients, and food groups, few have focused on young adults as in the present study. The relationship between serum homocysteine concentrations and vegetable and fruit intake was examined in a cohort of European adolescents, but no relationship between serum homocysteine concentrations and total vegetable and fruit intake was found [39]. However, that study did not examine vegetable and fruit intake separately, which may have led to different results from our study. In Japan, a study of young women that examined the relationship between serum homocysteine concentrations and nutrients and food groups and also examined the effects of vitamin B_2_, vitamin B_6_, vitamin B_12_, and folate has also been conducted [40]. However, the association of vitamins and minerals other than vitamin B_2_, vitamin B_6_, vitamin B_12_, and folate, as well as dietary fiber, with serum homocysteine concentrations was not examined. Consequently, it is possible that the relationships between serum homocysteine concentration and soluble fiber, insoluble fiber, and total dietary fiber, which were shown to be relevant in this study, may be a unique finding. Many studies on homocysteine concentrations, mainly in middle-aged and elderly subjects in Western countries, have been reported. The determinants of serum homocysteine concentrations may vary by country, culture, sex, age, and lifestyle, among others, and there may be differences in factors affecting homocysteine concentrations in different target populations. Further studies on young women in non-Western countries are needed, as there are few comparative studies available.

The strength of this study is that we conducted a dietary survey for 7 consecutive days, and the types and weights of individual foods consumed by the subjects were estimated and analyzed with a high degree of accuracy based on digital images and dietary records. However, this study had several limitations that should be considered when interpreting the results. First, given the cross-sectional nature of the study design, it was not possible to prove a causal relationship with serum homocysteine concentrations. Second, because the study was conducted at one university that specializes in nutrition and included only women, the study results might not be broadly applicable. Third, many factors are possibly involved in homocysteine metabolism, including lifestyle, but it is difficult to eliminate all confounding factors, so it is possible that some confounding factors remained in the analysis and influenced the results.

## 5. Conclusions

This study investigated the effects of nutrients other than vitamin B_6_, vitamin B_12_, and folate, which have been shown to be related to homocysteine metabolism, and other dietary factors on reducing blood homocysteine levels. The results showed that soluble fiber, insoluble fiber, and total dietary fiber were negatively associated with serum homocysteine concentrations. Among the food groups, higher intakes of fruits and mushrooms were found to have lowered serum homocysteine concentrations. A diet with higher fiber content may have contributed to lower serum homocysteine concentrations. These results indicate that fruits and mushrooms may be especially useful food sources. However, studies of young women in non-Western countries, such as the present study, are scarce, so it is important to conduct additional studies in non-Western populations.

## Figures and Tables

**Table 1:** Characteristics of the study population.

Variables		Overall (n = 227)
Age	(years)	20 [19, 21]
Height	(cm)	158.4 [155.3, 162.4]
Body weight	(kg)	51.4 ± 5.6
BMI	(kg/m^2^)	20.3 ± 1.9
Body fat percentage	(%)	25.4 ± 4.2
Mean systolic blood pressure	(mmHg)	107 [100, 115]
Mean diastolic blood pressure	(mmHg)	69 [64, 74]
Homocysteine	(µmol/L)	6.39 [5.53, 7.41]

$\bar{X}$ ± SD or median [25th, 75th percentiles].

**Table 2:** Relationship between energy/nutrient intake and serum homocysteine concentration.

		Amount of Intake	Model 1 ^¶^		Model 2 ^¶^		Model 3 ^¶^	
			βcoefficient	*p* Value ^§^	βcoefficient	*p* Value ^§^	βcoefficient	*p* Value ^§^
Energy	(kcal/day)	1729 [1454, 1961]						
Protein	(% energy)	14 [13, 16]	−0.196	0.0036	−0.154	0.0540	−0.102	0.2048
Fat	(% energy)	32 [29, 35]	0.053	0.4281	0.039	0.5618	0.010	0.8755
Carbohydrate	(% energy)	54 [51, 57]	0.027	0.6819	0.007	0.9142	0.018	0.7866
Protein	(g/1000 kcal)	35.3 [32.8, 38.8]	−0.201	0.0029	−0.159	0.0468	−0.113	0.1625
Fat	(g/1000 kcal)	35.6 [31.8, 39.1]	0.053	0.4268	0.039	0.5607	0.011	0.8735
Carbohydrate	(g/1000 kcal)	130 [122, 139]	−0.005	0.9424	−0.027	0.6890	−0.011	0.8646
Soluble dietary fiber	(g/1000 kcal)	2 [2, 2]	−0.286	<0.0001	−0.311	<0.0001	−0.281	0.0023
Insoluble dietary fiber	(g/1000 kcal)	5 [4, 6]	−0.241	0.0003	−0.255	0.0003	−0.195	0.0151
Total fiber	(g/1000 kcal)	7 [6, 8]	−0.268	<0.0001	−0.298	<0.0001	−0.255	0.0069
Vitamin A	(µg retinol activity equivalent/1000 kcal)	243 [197, 295]	−0.148	0.0270	−0.101	0.1655	0.034	0.6910
Vitamin D	(µg/1000 kcal)	2.3 [1.4, 3.3]	−0.130	0.0568	−0.054	0.5095	−0.062	0.4343
Vitamin E	(mg/1000 kcal)	3.8 [3.3, 4.4]	−0.144	0.0320	−0.130	0.0720	−0.089	0.2196
Vitamin K	(µg/1000 kcal)	118 [84, 155]	−0.225	0.0008	−0.211	0.0030	−0.125	0.1528
Vitamin B_1_	(mg/1000 kcal)	0.5 [0.4, 0.6]	−0.124	0.0719	−0.138	0.0950	−0.086	0.2995
Vitamin B_2_	(mg/1000 kcal)	0.6 [0.5, 0.7]	−0.154	0.0232	−0.121	0.1232	−0.049	0.5506
Vitamin B_6_	(mg/1000 kcal)	0.6 [0.5, 0.7]	−0.079	0.2500				
Vitamin B_12_	(µg/1000 kcal)	2.5 [1.6, 3.8]	−0.169	0.0137				
Folate	(µg dietary folate equivalents/1000 kcal)	143 [121, 170]	−0.231	0.0006	−0.241	0.0021		
Vitamin C	(mg/1000 kcal)	42 [32, 54]	−0.186	0.0054	−0.238	0.0047	−0.138	0.1649
Sodium	(mg/1000 kcal)	1908 [1668, 2208]	−0.005	0.9461	0.017	0.8064	0.035	0.6104
Potassium	(mg/1000 kcal)	1091 [963, 1230]	−0.224	0.0008	−0.259	0.0015	−0.169	0.0980
Calcium	(mg/1000 kcal)	255 [224, 306]	−0.221	0.0009	−0.202	0.0033	−0.133	0.0838

n = 227, median [25th,75th percentiles]. ^§^; *p*-values were multiple regression analyses with serum homocysteine concentration as the objective variable and nutrient intake (per 1000 kcal) as the explanatory variable. ^¶^; Model 1 is adjusted for age, BMI, menstrual cycle, pill use, and physical activity; Model 2 is adjusted for Model 1 + vitamin B6 and vitamin B12 intake; Model 3 is adjusted for Model 2 + folate intake.

**Table 3:** Relationship of serum homocysteine concentration to intake of each food group.

	(g/day)	(g/1000 kcal)	(g/1000 kcal)	(g/1000 kcal)	*p* Value for Trend ^§^
		T1 (n = 75) ^¶^	T2 (n = 76) ^¶^	T3 (n = 76) ^¶^	
Cereals	344 [300, 397]	163 [150, 174]	200 [190, 211]	248 [234, 272]	0.7194
Tubers and roots	31 [19, 48]	9 [5, 11]	18 [16, 21]	32 [26, 39]	0.4418
Sugar and sweeteners	11 [6, 17]	3 [3, 4]	7 [5, 7]	11 [10, 13]	0.1902
Nuts	2 [1, 3]	0 [0, 0]	1 [1, 1]	3 [2, 4]	0.5505
Green and yellow vegetables	61 [41, 91]	21 [14, 26]	36 [32, 40]	58 [53, 68]	0.0548
Other Vegetables	100 [72, 132]	40 [30, 45]	57 [54, 63]	82 [74, 97]	0.1127
Fruits	49 [16, 92]	7 [3, 11]	29 [23, 36]	61 [49, 83]	0.0060
Mushrooms	8 [3, 15]	1 [0, 2]	5 [4, 6]	11 [8, 14]	0.0091
Seaweed	3 [1, 7]	0 [0, 1]	2 [1, 2]	5 [4, 7]	0.3715
Pulses	29 [15, 50]	6 [2, 8]	17 [15, 19]	36 [26, 53]	0.0929
Seafood	32 [17, 53]	8 [4, 11]	18 [15, 22]	34 [30, 42]	0.0573
Meat	66 [44, 90]	24 [20, 28]	38 [34, 43]	56 [52, 65]	0.1804
Egg	33 [19, 49]	9 [5, 12]	19 [16, 22]	31 [26, 36]	0.0743
Dairy products	103 [62, 166]	29 [20, 38]	60 [51, 65]	107 [96, 128]	0.1837
Fats and oils	11 [8, 15]	4 [3, 5]	7 [6, 7]	9 [8, 10]	0.6495
Confectioneries	30 [14, 50]	5 [2, 9]	18 [15, 21]	35 [28, 44]	0.9064
Beverages	283 [141, 415]	63 [33, 84]	158 [133, 176]	287 [230, 360]	0.8447
Seasonings & Spices	41 [30, 53]	17 [14, 19]	23 [22, 25]	34 [29, 41]	0.1787

n = 227, median [25th, 75th percentiles]. ^¶^; subject tertiles differ by food group. ^§^; analysis of covariance was performed using the tertiles of intake by food group (g/1000 kcal) and serum homocysteine concentration as the objective variable. Adjusted for age, BMI, menstrual cycle, pill use, physical activity, and vitamin B_6_, vitamin B_12_, and folate intake.

**Table 4:** Dietary fiber intake by food group (mg/1000 kcal).

	Soluble Dietary Fiber	Insoluble Dietary Fiber	Total Fiber
Cereals	440 [311, 599]	1271 [1061, 1614]	1710 [1411, 2227]
Tubers and roots	105 [51, 180]	283 [153, 461]	395 [220, 629]
Sugar and sweeteners	0 [0, 0]	0 [0, 0]	0 [0, 0]
Nuts	11 [5, 21]	84 [31, 154]	98 [38, 169]
Green and yellow vegetables	237 [173, 334]	758 [532, 1056]	1007 [705, 1374]
Other Vegetables	340 [260, 452]	821 [645, 1055]	1190 [922, 1517]
Fruits	125 [49, 231]	287 [123, 520]	430 [178, 765]
Mushrooms	11 [4, 22]	213 [84, 377]	221 [88, 391]
Seaweed ^§^	–	–	189 [73, 320]
Pulses	111 [25, 213]	291 [81, 601]	388 [119, 826]
Seafood	0 [0, 0]	0 [0, 0]	0 [0, 0]
Meat	0 [0, 13]	0 [0, 40]	0 [0, 53]
Egg	0 [0, 0]	0 [0, 0]	0 [0, 0]
Dairy products	0 [0, 4]	0 [0, 0]	0 [0, 4]
Fats and oils	0 [0, 0]	0 [0, 0]	0 [0, 0]
Confectioneries	95 [36, 174]	179 [69, 357]	291 [106, 531]
Beverages	5 [0, 36]	3 [0, 77]	20 [0, 115]
Seasonings & Spices	48 [28, 70]	189 [107, 278]	243 [142, 346]

n = 227, median [25th, 75th percentiles]. ^§^; No data on dietary fiber intake from seaweeds because soluble and insoluble fiber are not listed in Japan’s Standard Tables of Food Composition, 2015.

## Data Availability

The data sets used and analyzed in this study are available from the Kagawa Nutrition University upon reasonable request.
